# Comparison of antibiotic prescription indexes by patient and physician gender and age: a cross-sectional study based on administrative data

**DOI:** 10.1186/s12875-026-03221-9

**Published:** 2026-03-03

**Authors:** Lorenzo Stacchini, Silvia Forni, Tommaso Manciulli, Guglielmo Bonaccorsi, Leonardo Grilli, Flavia Franconi, Fabrizio Gemmi

**Affiliations:** 1 Department of Health Sciences, University of Florence, Florence, Italy; 2 Quality and Equity Unit, Regional Health Agency of Tuscany, Florence, Italy; 3 Department of Statistics, Computer Science, Applications, University of Florence, Florence, Italy; 4 Laboratory of Gender- Medicine, National Institute of Biostructures and Biosystems, Sassari, 07100 Italy

**Keywords:** Antimicrobial resistance, Defined daily doses, Antimicrobial consumption, Gender differences, Intersectional studies

## Abstract

**Background:**

Few studies have examined the association between gender-differentiated patient–physician interactions and antimicrobial prescription (AP) patterns. We analyzed gender-specific differences in AP in relation to the sex and age of general practitioners (GPs), drawing on data from a comprehensive regional database in Tuscany, Italy.

**Methods:**

We extracted data on primary care APs for 2023 using a population sample of 3,022,332 patients and 2,311 GPs. We used defined daily doses per 1,000 population per day (DDD/1000 population/day), the percentage of subjects with at least one antimicrobial prescription (AP prevalence), and the percentage of Access AP according to Access, Watch and Reserve (AwaRE) classification as indicators. We fitted regression models for each indicator with patient-physician gender dyads and other covariates. Adjusted indicators and average marginal effects (AMEs) of having a female GP separately for the female and male patients are reported together with the difference between the above AMEs.

**Results:**

A total of 1,583,893 female patients (52.4%) and 1,104 female GPs (54.1%) were included. Same-sex care was observed for 774,411 female patients (48.9%) and 815,887 male patients (56.7%). Female GPs prescribed fewer antibiotics—in terms of both DDD/1000 population/day and prevalence of AP—and were more likely to prescribe Access antibiotics. These differences were more pronounced among male patients. Among male patients, having a female GP was associated with an adjusted AME of -0.78 DDD/1000 population/day (95% CI -1.09 to -0.47), -0.21% in AP prevalence (95% CI -1.74 to -0.69), and + 0.73% in proportion of Access AP (95% CI -0.10 to 1.56). Among female patients with a female GP, the adjusted AME was − 0.51 DDD/1000 population/day (95% CI -0.86 to -0.15), -0.59% in AP prevalence (95% CI -1.18 to -0.004), and + 0.24% in proportion of Access AP (95% CI -0.52 to 0.99). When the patient-GP gender dyad and GP age were included, male GPs under 40 years of age showed consistently lower antibiotic prescription—both in DDD/1000 population/day and AP prevalence—and a higher proportion of Access AP compared with female GPs, particularly among female patients. In this subgroup, the adjusted AME of having a female GP was + 0.87 DDD/1000 population/day (95% CI 0.06 to 1.69), + 1.86% in AP prevalence (95% CI 0.52 to 3.2), and − 1.44% in Access AP (95% CI -3.01 to 0.13).

**Conclusions:**

In our analysis of a large administrative dataset, we observed that the gender-differentiated interaction between physician and patient was associated with antimicrobial prescription. Overall, female GPs showed lower AP rates. However, among physicians under 40 years of age, male GPs had lower prescription indicators than their female counterparts, especially for male patients. The differences highlighted in our study could be the target of stewardship interventions.

## Introduction

Antimicrobial resistance (AMR) is an increasingly important public health problem, with an estimated global mortality of 4.95 million [1]. In 2017, the World Bank estimated the costs of AMR to be one trillion dollars [2]. Optimizing antibiotic consumption (AC) is key to reducing AMR [1]. European countries monitor the quantity and appropriateness of AC using indicators such as defined daily doses (DDD) and the Anatomical Therapeutic Chemical (ATC) system. Appropriateness indicators are defined by ECDC and National Action Plans [3–5]. The WHO has adopted the Access, Watch, and Reserve (AWaRe) classification, dividing antibiotics into three categories to facilitate stewardship and monitoring efforts [6]. While classification was intended as a tool to guide prescription with resistance and stewardship considerations in mind, quality indicators derived from this classification have been adopted by the ECDC and national action plans. Italy has a higher AC than the European average, with prescription patterns differing between regions [4]. In Italy, outpatient prescriptions by General Practitioners (GPs) account for around 68% of all prescriptions [3]. Tuscany has lower DDD/ 1000 population/day (15.4) and Access prescription rates compared to southern regions of Italy and in line with the global trend, which shows a north-south gradient in both the rate of Access prescriptions and DDD-based indicators [3].

Studies have shown that patient gender is a determinant of physician practice and patient outcomes, such as in-hospital mortality and the probability of receiving delayed care [7–12]. However, there is little research on the influence of patient gender on antibiotic prescription (AP) [12]. Integrating data on sex or gender with social determinants (i.e. using an intersectional approach) to study AP and AMR has been attempted in other studies [13]. However, only one study focused on patient-physician dyads: a study conducted in the Netherlands on sore throat symptoms analyzed the role of sex concordance between GP and patient and their interaction in antibiotic prescription behavior, finding that female doctors prescribed fewer antibiotics to both female and male patients [9]. The objective of this study was to evaluate whether patient and physician gender may have a role in influencing the probability of antimicrobial prescription, and whether other factors (age, clinical and socioeconomic determinants) may act as covariates in the GP decision-making process.

## Methods

### Study design and population

This is a cross-sectional study. Our patient population (and therefore, GP population) included individuals who were: (i) residents in Tuscany between January and December 2023; (ii) over the age of 15. We excluded GPs (and their respective patients) with fewer than 100 assigned patients to eliminate GPs who were at the start or end of their careers. Additionally, following a previously published study, we excluded pediatric patients, defined as individuals under 16 years of age at the time of data extraction on January 1, 2024 [9]. In pediatric consultations, the role played by parents or legal guardians makes direct comparisons with adult patients challenging [9]. However, we were unable to determine whether a caregiver was involved in antimicrobial prescription. We were also unable to compare results between dyads based on prescription indications, as this information is not included in the dataset due to privacy reasons and lack of a standardized reporting system for indications. Our analysis included only AP by GPs and reimbursed by the Italian National Health Service (SSN). Therefore, antibiotics purchased privately by patients without SSN reimbursement were not captured in the dataset. Furthermore, our data refer to prescribed medications only, which were not necessarily dispensed or consumed, as outpatient pharmacy records were not available. In Italy, patients can choose their GP from a list of doctors managed by a local health authority (ASL). Each patient is assigned one GP, with exceptions for short-term substitutions. Tuscany has three ASLs whose data is fed into a centralized surveillance system for antimicrobial prescriptions. This system enables each prescription to be traced to a specific GP-patient dyad.

### Study variables

For each patient-physician pair, we considered the following characteristics: (i) age and gender of GP; (ii) age and gender of patient; (iii) patient Multisource Comorbidity Score (MCS); (iv) deprivation index (DI) from the census block of residence; (v) urbanization level of the municipality of residence.

The Multisource Comorbidity Score (MCS) is a prognostic score that has been shown to predict all-cause mortality [14]. It is derived from hospital discharge records and medication prescription data. For each individual in the study population, the presence of 34 diseases or conditions is identified using inpatient diagnostic codes and records of drugs dispensed in the preceding two years. The MCS for each individual is then calculated as the weighted sum of these conditions. In this study the resulting score was then categorized into three levels representing increasing comorbidity burden.

The DI, which includes five socio-economic indicators [15, 16] was calculated at census block of residence and used as a proxy for individual socio-economic status. The Italian DI is based on data from the national population census and is a comprehensive measure of disadvantage in the possession of social and material resources among residents in each census block, similar to neighborhoods. The DI is calculated using five indicators that collectively represent the complexity of social and material deprivation: low education level, unemployment, lack of home ownership, single-parent households and overcrowding. The DI was calculated for each census block as the sum of the standardized socioeconomic indicators derived from the national population census. Each individual was assigned the DI value corresponding to their area of residence. The DI was then divided into quintiles based on its distribution in the study population.

Urbanization was defined according to the guidelines of the Italian National Institute of Statistics which categorizes areas as urban, suburban, rural, peripheral rural and very-peripheral rural areas.

For the calculation of population per day, all individuals were assumed to contribute 365 days, except those who died during the study period, whose days at risk were calculated from January 1, 2023 to their date of death.

### Prescription indicators

The AP indicators used for the study are reported in Table 1. These indicators were selected based on their availability through data gathered by the Regional Health Authority and because of their use in Italy’s National Action Plan or by the Italian National Drug Agency [3, 4].

**Table 1:** Indicator definitions

Indicator	Definition	Type	Target
Defined Daily Doses per 1000 population per day (DDD/1000 population/day)	Antibiotic usage (ATC group J01) in defined daily doses per 1000 population per day.	Usage	Decrease
Prevalence of AP x 100	Percentage of subjects with at least one antimicrobial prescription (AP) in a calendar year.	Usage	Decrease
Rate ofAccess AP x 100	Ratio between Access AP and total prescriptions in DDD according to the AWaRe classification (ECDC standard ≥ 65%).	Prudent use of antibiotics	Increase

*DDD* defined daily doses, *AP *antimicrobial prescription, *ATC *Anatomical therapeutic Chemical. Targets based on expected direction as reported in italy’s National action plan or by the Italian National drug agency [3, 4]

### Data collection

We integrated pharmaceutical prescription records and hospital discharge records with the civil registry archive. The civil registry archive includes a unique pseudonymized identifier, sex, birth date, municipality, and a unique identifier of the assigned GP. The DI and urbanization level were assigned based on the municipality of residence. The pharmaceutical prescription data include drugs prescribed by both public and private pharmacies within the regional territory upon presentation of a medical prescription. Each record includes the unique pseudonymized identifier and the ATC code DDD for the prescribed drug molecules. The hospital discharge records include the unique pseudonymized identifier and six diagnostic ICD-9-CM codes. Hospital discharge records and pharmaceutical prescription data covering the three years preceding the study period were used to compute the MCS. The data linkage between these three registries was established through the unique pseudonymized identifier, creating the database used for our analysis.

### Statistical analysis

We conducted a descriptive analysis for each indicator and all mentioned variables. The associations between gender-based patient-physician dyads and indicators were tested using a zero-inflated negative binomial regression model [17] for the DDD with days at risk as exposure (the same covariates were used in the count and in the inflation equation), which accounted for overdispersion and excess zeros in the distribution. A logistic regression model was used for the AP prevalence data, while a fractional logistic regression approach was applied to the rate of Access AP. All models utilized cluster-robust standard errors to consider data clustering at the GP level. The regressions compared sex-based patient-physician dyads and were adjusted for patient characteristics (age, MCS, DI, ASL, urbanization level) and physician characteristics (age). Patient age was categorized into four brackets (15–34, 35–54, 55–74, and ≥ 75 years), while GP age was grouped into 5-year ranges (< 35, 35–39, 40–44, 45–49, 50–54, 55–59, 60–64, and ≥ 65 years). To investigate whether the effect of the patient-physician gender dyad on the indicators varied across patient characteristics, separate models were fitted with one interaction term at a time between the dyad and each covariate. Similarly, by adding an interaction term, we explored whether the effect of the patient-physician gender dyad varied by GP age. Adjusted indicators for each patient-physician gender dyad were estimated using marginal effects [18]. To determine whether the effect of having a female GP differed by patient gender, we calculated the average marginal effects (AMEs) of having a female GP separately for female and male patients by estimating contrasts of margins. The gender-related difference was quantified as the difference in AMEs between female and male patients. We reported all estimates with a 95% confidence level. Data were prepared using SQLDeveloper, version 22.2 (Oracle Corporation, USA), and analyses were conducted using Stata, version 17 (StataCorp, USA).

### Ethical considerations

This was a retrospective study based on the linkage among regional healthcare pseudonymization databases. According to Italian Legislative Decree No 196/2003 (Personal Data Protection Code) and Regulation (EU) 2016/679 (GDPR*)* on the protection of personal data, ethical approval and informed consent were not required for this study. The study adhered to the principles of the Declaration of Helsinki.

## Results

### Physician and patient demographics, clinical data 

Out of the 2,311 GPs included in the study, 1,207 were male (52.2%). Male GPs were significantly older (58.6, SD 11.7 years) than their 1,104 female (51.0, SD 11.7 years) counterparts. Male doctors had a higher number of patients (mean 1,346.0, SD 361.3) than their female colleagues (1,265.0 SD 392), *p* < 0.001.

The GP population included in the study served 3,022,332 patients (93.1% of the total regional population at the time of the study). Of these, 1,583,893 (52.4%) were female patients. The overall concordance between patient and GP gender was 52.6%, with 774,411 female patients assigned to same-sex GPs (48.9%) and 815,887 male patients assigned to same-sex GPs (56.7%). The overall mean patient age was 53.1 (19.8 SD). Male GPs cared for older patients, with the largest difference in female patients (Table 2). Analyzing the MCS and DI distribution, we found that the only significant difference was that female GPs assisted a higher proportion of healthier female patients (Table 2). Although statistically significant, the differences regarding the distribution of DI by patient and GP gender were minimal (Table 2). Among patients of male GPs, a higher proportion of patients resided in rural areas, regardless of gender.

**Table 2:** Socio-economic, geographical and clinical data by patient–physician gender dyad

Patient group	Female patients			Male patients		
Physician group	Female physician	Male physician	Mean difference	Female physician	Male physician	Mean difference
Patients, *n*	774,411	809,482		622,552	815,887	
Mean age (SD), years	53.10 (20.30)	55.42 (19.78)	2.33*	51.60 (19.61)	51.87 (19.39)	0.27*
Multisource Comorbidity Score (*n*, %)						
0	475,941 (61.46%)	477,958 (59.04%)	2.41*	417,608 (67.08%)	547,471 (67.10%)	-0.02
1–4	210,390 (27.17%)	229,835 (28.39%)	-1.22*	138,809 (22.29%)	182,058 (22.31%)	-0.02
5–9	55,482 (7.16%)	64,392 (7.95%)	-0.79*	39,243 (6.30%)	51,734 (6.34%)	-0.04
≥ 10	32,598 (4.21%)	37,297 (4.61%)	-0.39*	26,892 (4.32%)	34,624 (4.24%)	-0.07*
Deprivation Index (DI) (*n*, %)						
1- Less deprived	143,763 (18.56%)	148,070 (18.29%)	0.27*	114,461 (18.38%)	147,014 (18.01%)	0.37*
2	130,198 (16.81%)	132,099 (16.31%)	0.49*	104,075 (16.71%)	131,205 (16.08%)	0.64*
3	127,641 (16.48%)	129,667 (16.01%)	0.46*	101,520 (16.30%)	129,031 (15.81%)	0.49*
4	124,927 (16.13%)	134,104 (16.56%)	-0.43*	100,059 (16.07%)	134,693 (16.50%)	-0.44*
5 - Most deprived	131,458 (16.98%)	136,756 (16.89%)	-0.08	104,260 (16.74%)	140,430 (17.21%)	-0.46*
* Missing*	116,424 (15.03%)	128,786 (15.90%)	-0.87*	98,177 (15.77%)	133,514 (16.36%)	-0.59*
Residence classification (*n*, %)						
Urban	352,968 (45.58%)	368,494 (45.52%)	0.06	270,153 (43.39%)	368,918 (45.22%)	-1.82*
Suburban	211,591 (27.32%)	199,324 (24.62%)	2.70*	174,589 (28.04%)	202,869 (24.86%)	3.20*
Rural	209,852 (27.1%)	241,664 (29.85%)	-2.76*	177,810 (28.56%)	244,100 (29.92%)	-1.36*
Local Health Authority (*n*, %)						
Center	347,553 (44.88%)	338,193 (41.78%)	-3.10%*	273,040 (43.86%)	346,362 (42.45%)	-1.40%*
North-West	246,861 (31.88%)	296,614 (36.64%)	4.76%*	199,316 (32.02%)	294,929 (36.15%)	4.13%*
South-East	179,997 (23.24%)	174,675 (21.58%)	-1.66%*	150,196 (24.13%)	174,596 (21.40%)	-2.72%*

* Indicates significant differences between patient dyads

### Overall trends of antibiotic prescription

The unadjusted AP rate expressed as DDD per 1000 population per day was 16.57 (95% CI 16.57 to 16.58). Female patients had a rate of 17.87 (95% CI 17.85 to 17.88) compared to 15.15 (95% CI 15.14 to 15.16) DDD/1000 population/day for males. After adjusting for covariates, female GPs prescribed lower quantities of antibiotics to both sexes compared to male GPs. The most significant difference was seen in male patients, with an AME of -0.78 DDD/1000 population/day, (95% CI -1.09 to -0.47) while in female patients the AME was − 0.51 DDD/1000 population/day, 95% CI -0.86 to -0.15. The reduction in quantities prescribed by female GPs was more pronounced for male patients with a difference in AMEs of 0.27 DDD/1000 population/day, 95% CI 0.07 to 0.47) (Table 3). We thus estimated that female GPs prescribe 391,382 fewer DDDs per year in Tuscany (-2.2% of total DDDs), based on AMEs by patient-GP gender. Overdispersion for the DDD per 1000 population per day indicator was verified via the alpha (α) parameter in the zero-inflated negative binomial model. In the model used for the estimates reported in Table 1, the estimated overdispersion parameter was α = 0.67 (95% CI: 0.66–0.67), indicating significant overdispersion relative to the Poisson distribution.

**Table 3:** Adjusted indicators by patient–physician sex dyads

	Adjusted rate (95% CI)		AME	Difference-in-AMEs (95% CI)
	Female physician	Male physician	(95% CI)	
DDD per 1000 population per day				
Female patients	17.68 (17.44 to 17.93)	18.19 (17.93 to 18.44)	− 0.51 (-0.86 to − 0.15)	0.27 (0.07 to 0.47)
Male patients	15.49 (15.28 to 15.69)	16.27 (16.04 to 16.49)	− 0.78 (-1.09 to − 0.47)	ref.
Prevalence of AP x 100				
Female patients	40.11 (39.72 to 40.51)	40.70 (40.29 to 41.11)	− 0.59 (-1.18 to -0.004)	0.62 (0.37 to 0.87)
Male patients	33.37 (33.03 to 33.71)	34.59 (34.22 to 34.96)	-1.21 (-1.74 to − 0.69)	ref.
Rate of Access AP x 100				
Female patients	48.91 (48.4 to 49.41)	48.67 (48.12 to 49.21)	0.24 (-0.52 to 0.99)	− 0.49 (-0.89 to − 0.09)
Male patients	52.92 (52.36 to 53.47)	52.19 (51.59 to 52.79)	0.73 (-0.1 to 1.56)	ref.

*AP* antimicrobial prescription, *DDD D*efined daily doses, *AME *average marginal effect

The unadjusted prevalence of subjects with at least one AP was 37.41% (95% CI 37.35% to 37.46%), Female patients had a higher prescription rate (41.12%, 95% CI 41.0% to 41.19%) compared to males (33.33%, 95% CI 33.25% to 33.40%). After adjustment, having a female GP led to lower prevalence rates for both female patients (AME =-0.59%, 95% CI -1.18% to 0.004%) and male patients (AME =-1.21%, 95% CI -1.74% to -0.69%). This reduction was more significant for male patients with a difference in AME of 0.62% (95% CI 0.37% to 0.87%) (Table 3).

Regarding the rate of Access AP, the overall unadjusted rate was 49.72% (95% CI 19.69% to 49.76%). Women had a lower Access rate (49.17%, 95% CI 49.12% to 49.21%) than men (50.44%, 95% CI 50.39% to 50.49%). In both cases, patients fell below the European standard of at least 65% Access prescriptions. After adjustment, female GPs prescribed a greater proportion of Access molecules to both sexes, with a greater difference in male patients (AME = 0.73%, 95% CI -0.10% to 1.55%) than in female patients (AME = 0.24%, 95% CI -0.52% to 0.99%), resulting in a difference between AMEs of -0.49% (95% CI -0.89% to -0.09%) (Table 3).

**Fig. 1:**
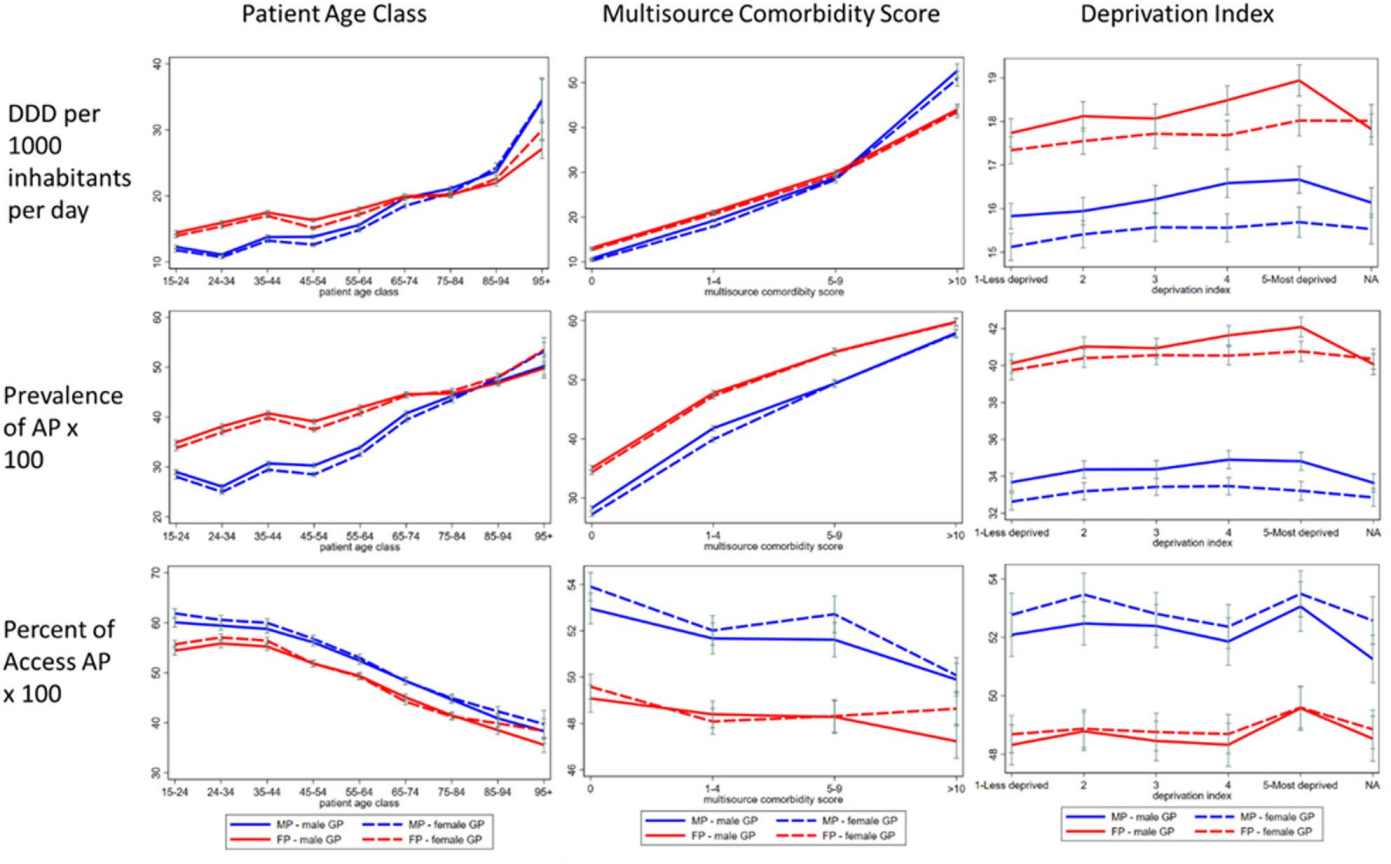
Adjusted indicators across patient age, comorbidity, and deprivation strata, by patient–physician gender dyad. AP= antimicrobial prescription, DDD= defined daily doses, FP= female patients, MP= male patients

**Fig. 2:**
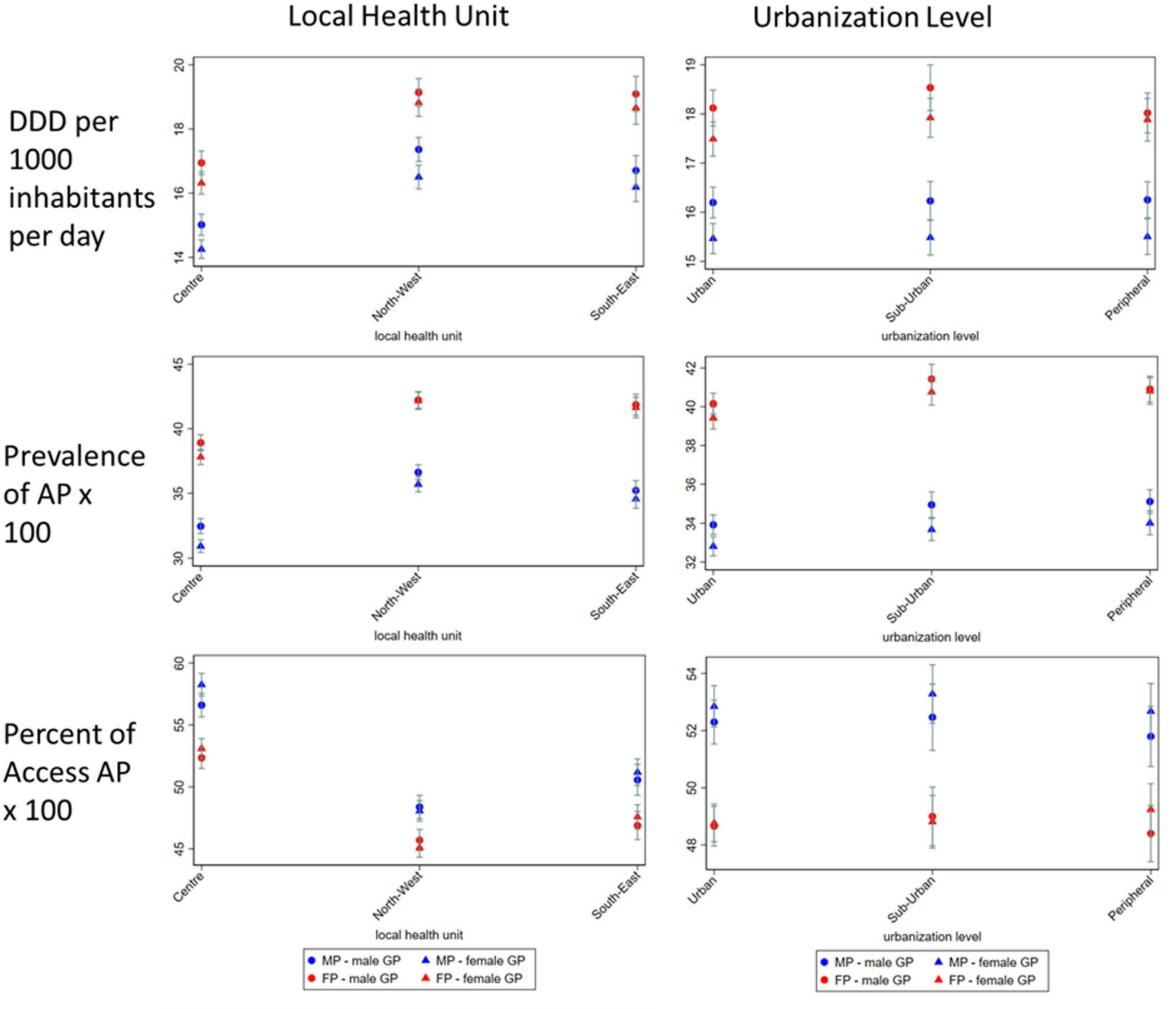
Adjusted indicators across local health authority and urbanization level strata, by patient–physician gender dyad. AP= antimicrobial prescription, DDD= defined daily doses, FP= female patients, MP= male patients

Adjusted indicators by patient–physician gender dyad and patient characteristics (Figs. 1 and 2) were estimated as marginal effects from models including interaction terms. Results were consistent across subgroups defined by patient age, deprivation index, MCS, level of urbanization, and ASL, confirming the robustness of the observed associations.

### Role of physician age

**Fig. 3:**
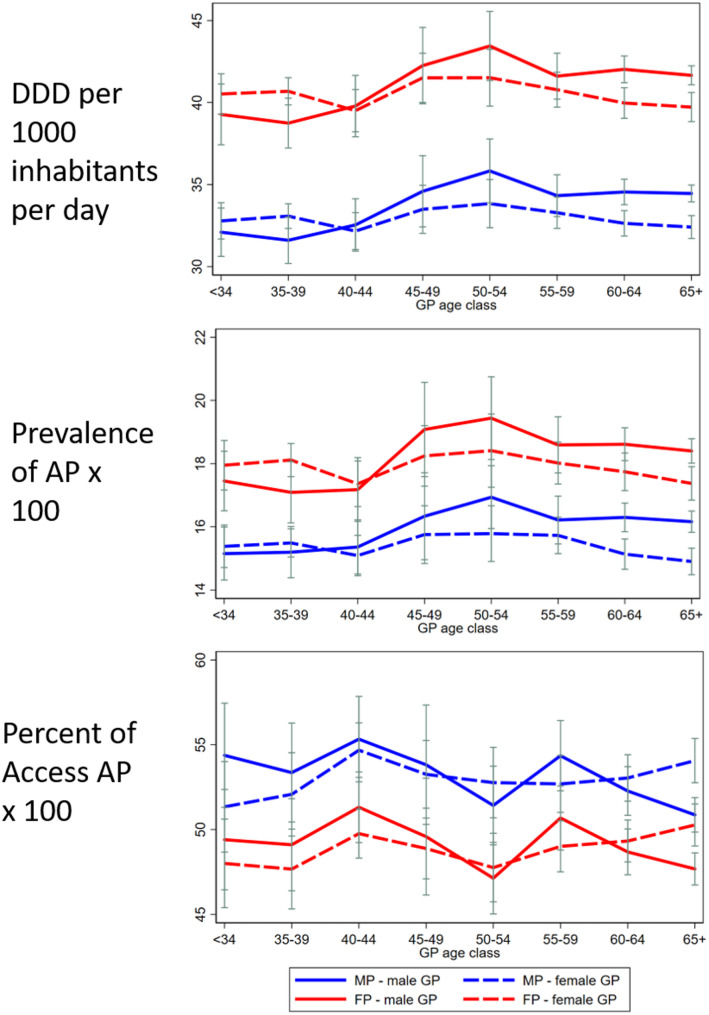
Adjusted indicators across GP age class by patient–physician gender dyad. AP= antimicrobial prescription, DDD= defined daily doses, FP= female patients, MP= male patients

After stratifying by physician age, the effect of the dyad varied, showing an inversion of trends between age ranges 35–39 and 40–44 (Fig. 3) therefore, an interaction term between the patient–physician gender dyad and GP age (< 40 vs. ≥40 years) was included in the model.

**Fig. 4:**
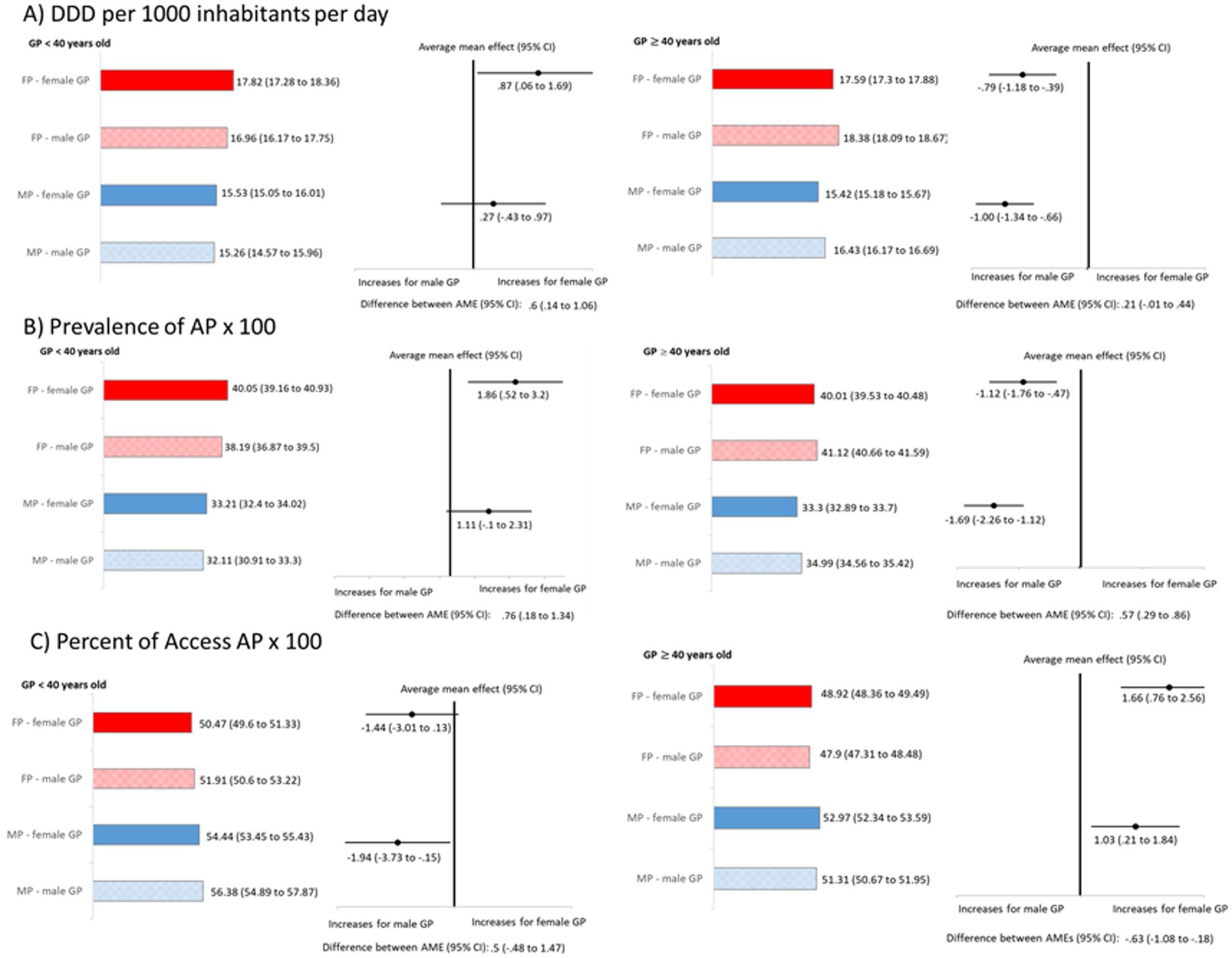
Adjusted indicators across two GP age groups (GPs aged ≤ 40 years old on the left and > 40 on the right side) by patient–physician gender dyad. The figure is organized in three panels representing: **A**) DDD per 1,000 population per day; **B**) Prevalence of AP x 100; **C**) Percent of Access AP x 100. For each panel, bars represent indicator estimates with 95% CI. Dots indicate the AMEs for each gender dyad, reported with direction of the effect and difference between AMEs. AP= antimicrobial prescription, DDD= defined daily doses, FP= female patients, MP= male patients

Among GPs under 40, DDD per 1,000 population per day was lower among male GPs, but this difference did not reach statistical significance for male patients. The difference was more evident for female patients (difference between AMEs = 0.87 DDD/1,000 population/day; 95% CI 0.06–1.69). Results were similar for AP prevalence (Figs. 3 and 4). The rate of Access AP was higher among male GPs, although the difference by physician gender was statistically significant only for male patients (AME for male patients: -1.941%; 95% CI -3.73% to -0.15%) (Fig. 4).

Conversely, among GPs aged 40 or older, both the DDD/1,000 population/day and the prevalence of AP were higher for male than for female GPs, for both male and female patients. Considering the AP prevalence indicator, the AME was significantly greater among male patients than among female patients (difference between AMEs = 0.57%; 95% CI 0.29% to 0.86%). Moreover, older male GPs prescribed a lower proportion of Access antibiotics compared with female GPs, particularly for female patients (difference between AMEs = -0.63%; 95% CI -1.08% to -0.18%) (Fig. 4).

## Discussion

To our knowledge, this is one of only two studies [9] examining the association between patient-physician gender dyads and AP. Many studies have observed that AP, and to some extent antibiotic consumption, is higher in women than in men, as shown by a systematic review and meta-analysis published in 2016, which highlighted that the effect of gender was influenced by age: women received more antibiotics than men between 16 and 74 years old while men aged 75 and older received more antibiotics [19]. Similar results were found in a large study from the UK, where, however, authors noted that indications affected prescription patterns and interacted with sex and age [20]. In the previously cited systematic review, the effect of gender in AP was lower in urban than in rural areas and decreased with physician age [19]. The authors of two systematic reviews, however, noted that the effects of patient and physician gender were heterogeneous and varied based on setting. These data seem to indicate that the prescriber’s gender and patient characteristics may be associated with changes in AP in some contexts [19, 21]. Regarding the study setting, our analysis focused on GPs since most AP occurs in primary care, which is also the setting in which fewer measures are adopted to limit the emergence of AMR [22, 23]. We also incorporated variables taking into account social and geographical variations in this large region of Italy. To our knowledge, our study is the first in Italy using a large-scale dataset to examine the relationship between patient-physician gender dyad and AP in the setting of primary care.

Regarding the interaction of patient and physician gender, one study designed to examine this component focusing on APs for sore throats found that female GPs had a higher propensity to apply a wait-and-see approach in the management of this condition if the patient was female [9]. Another study in China found that male physicians had a higher propensity to prescribe antibiotics to their patients. In the same study, GPs with an overall better knowledge of AP (assessed through a questionnaire) performed better in rational antibiotic prescription, consistent with data from two systematic reviews [19, 21]. In the study in China, female GPs showed a lower propensity to prescribe antibiotics, although they did use antibiotic combinations more frequently [24]. An older study in Italy found similar associations, with male physicians prescribing more antibiotics and more frequently resorting to parenteral administration [25].

A reason for the positive effects on patient indicators for female-to-female dyads could be a better communication experience between patients and physicians, as reported in the literature [10, 24, 26].

Our results show that female GPs prescribed 391,382 DDDs x 1000 population per day less than their male counterparts (a reduction of -2.2% considering the total number of DDDs prescribed at the regional level). Some of the overall differences in the cohorts could play a role in determining AP. Our data suggest that increasing GP age is associated with increases in AP, and the male GPs in our study were significantly older. The fact that a larger proportion of patients living in rural areas are assigned to male patients may also play a role, although the differences observed but it did not appear to be significantly associated with indicators when accounted for in our model.

While, overall, female physicians tend to prescribe fewer antibiotics and more appropriately, factoring in physician age led to differences between the two age groups we defined, as male physicians aged under 40 old had lower prescription indicators than their female counterparts, although the differences were smaller than those observed in the older age cohort, and the clinical effect of such differences is uncertain.

Of relevance, the differences in GP age groups found in our study could not be tested against knowledge, attitude and practice (KAP) data as was done in these studies [9, 24]. One possible explanation for differences observed between age groups could be connected to the presence of a structured training program for GPs, which was introduced in 2007. Before the start of the program, GPs were recruited on a voluntary basis and no dedicated training program was in place. The GP training program is managed on a regional basis, and trainees are provided with notions of AMR and AP appropriateness. Of note, the previously cited study from the UK found that updates and guidelines did not positively affect prescription appropriateness, which could explain differences between trained and untrained physicians [20].

Another difference as compared to existing studies was the lack of data on AP indication (as these cannot be traced by the regional surveillance system used for this study). Most of the existing studies focus on upper respiratory tract infections and provide qualitative data or quantitative data based on a relatively low number of patients and physicians [21, 25]. One limitation connected to the nature of the dataset is the impossibility of ascertaining whether APs corresponded to ACs, although this should be the rule in Italy (i.e. a GP should only prescribe antibiotics that are to be consumed) this may not always be the case. We also have no data on the private purchase of antibiotics by patients. These can either be over the counter purchase of ATC-coded J01 drugs, which is in theory not allowed in Italy but has been found to occur in Italy and in Europe [27, 28]. Furthermore, data on antibiotic prescriptions not covered by the Italian National Health system (e.g. issued by private specialists and paid for by the patients) were also not available. In principle, all prescriptions issued by GPs for J01 drugs should fall within the coverage of the Italian SSN, ensuring the reliability of our dataset. However, the possibility of acquiring antibiotics privately may cause a decrease in requests to GPs, which are a key driver in AP [24]. However, given the large scale of our dataset, we believe that this issue does not constitute a particular bias in our study. Furthermore, we could not exclude the presence of a selection bias connected to the fact that patients in Italy can freely choose their doctor within a given geographical area, and sex concordance has been shown to play a consistent role in physician selection [10]. Another study on gender dyads and medical outcomes was able to eliminate this bias by working with hospitalists, whose patient allocation follows a quasi-random pattern, something that was not possible in our study [10].

Despite its limitations, our study had the merit of using a large-scale, real-life dataset that examines widely used indicators for the surveillance of AP. Another strength was accounting for clinical and socio-economic factors. These are known to have a role in medical decisions, and to thus interact with gender [10]. The lack of significant differences between urban and rural settings, as well as the absence of differences in MCS values in the patient-physician dyad, underscores the generalizability of our results to the Italian context.

Overall, our data provide insights into the relationship between physician and patient gender in the field of AP. Further studies on KAP in Italy are needed to better understand factors connected to the differences observed in our study. Furthermore, our data provide initial information that could potentially guide stewardship measures in our setting.

## Data Availability

The Regional Health Agency of Tuscany (ARS) acts as the data controller of the data used in this study, pursuant to Regional Regulation of Tuscany of 26 October 2021, No. 37/R (Annex A, Sheet No. 12). Anonymized data may be shared only provided that there is no risk of re-identification of individuals, in accordance with the guidance of the Italian Data Protection Authority (https://www.garanteprivacy.it). Thus, the data underlying this study are available on request once collapsed. Data access requests should be addressed to the corresponding author.
